# The Benefits of Receiver Clock Modelling in Satellite Timing

**DOI:** 10.3390/s21020466

**Published:** 2021-01-11

**Authors:** Weijin Qin, Xiao Wang, Hang Su, Zhe Zhang, Xiao Li, Xuhai Yang

**Affiliations:** 1 National Time Service Center, Chinese Academy of Sciences, Xi’an 710600, China; wangxiao@ntsc.ac.cn (X.W.); suhang@ntsc.ac.cn (H.S.); zhangzhe@ntsc.ac.cn (Z.Z.); lixiao202@mails.ucas.ac.cn (X.L.); yyang@ntsc.ac.cn (X.Y.); 2 Key Laboratory of Precise Positioning and Timing Technology, Chinese Academy of Sciences, Xi’an 710600, China; 3 School of Astronomy and Space Science, University of Chinese Academy of Sciences, Beijing 100049, China

**Keywords:** timing, stochastic clock model, PPP, kinematic scheme, static scheme

## Abstract

Satellite timing is an effective and convenient method that has been widely accepted in the time community. The key to satellite timing is obtaining a clean receiver clock offset. In this paper, instead of regarding the receiver clock offset as white noise, a two-state stochastic clock model involving three kinds of noise was conceived and used in PPP filter estimation. The influence of clock type and sampling time on satellite timing performance was first analysed. In addition, the kinematic scheme and static scheme were both investigated for meeting the demands of multi-occasional users. The values show that the model works well for both the kinematic scheme and static scheme; in contrast to that of the white noise model, the timing stability is enhanced at all the sampling times. For the six stations, especially when the averaging time is less than 1000 s, the average stability improvement values of the kinematic scheme are 75.53, 43.24, 75.00, 69.05, 40.57, and 25.45%, and the average improvement values of the static scheme are 65.49, 77.94, 56.71, 60.78, 64.41, and 39.41%. Furthermore, the enhancement magnitude is related to clock type. For a high-stability clock, the improvement of the kinematic scheme is greater than that of the static scheme, whereas for a low-stability clock, the improvement of the kinematic scheme is less than that of the static scheme.

## 1. Introduction

State-of-the-art techniques for remote time and frequency transfer, such as optical fibre links and quantum techniques, have been remarkably advanced and are being developed to meet the requirements of ultra-stable optical clock comparisons [1,2]. However, especially over intercontinental distances, optical fibre links and quantum techniques create challenges. GNSS-based time and frequency transfer are dependent on strict modelling of both code and carrier phase measurements, which can lead to precise measurements at sub-nanoseconds without being restricted by distance or weather conditions [3,4,5,6,7]; therefore, GNSS transfer is still the optimal method for use by high-demand time/frequency users.

Atomic clocks provide frequency signals to satellite navigation systems. How atomic clock information can be used to improve Position & Navigation &Timing (PNT) accuracy is our ongoing interest. In the past, clock modelling was rarely applicable to the user end due to its inferior performance. Benefitting from new technique developments, the considerably increased frequency stability of ground clocks makes clock modelling a reality. To date, a total of 100 stations located in the International GNSS Service (IGS) network are equipped with a high-precision atomic clocks (ftp://igscb.jpl.nasa.gov/igscb/station/general/loghist.txt). The atomic clocks operated in the tracking stations are generally classified into three types: the caesium atomic clock, rubidium atomic clock, and hydrogen maser. Clock modelling is not a new topic in PPP processing strategies. In 2018, Shi et al. [8] achieved multi-GNSS satellite clock estimation with an oscillator noise model in the presence of data discontinuities. In 2015, Thomas et al. [9,10] presented an extended Kalman filter that is effective for kinematic positioning. In 2013, Wang et al. [11] demonstrated the feasibility of the stochastic clock parameter with constrained variance in subsequent epochs, and the extent of the improvement was different for different types of atomic clocks. In 2018, Ge et al. [12,13] contributed the receiver clock offset model considering the correlation of the receiver clock offsets among adjacent epochs using an a priori value, which is suitable for single GNSS and multiple GNSS. In 2014, Lyu et al. [14] achieved covariance with white frequency modulation (WFM) noise, random walk frequency modulation (RWFM) noise and the correlation between the clock and ambiguity parameter and obtained better results in mitigating day boundaries. Some scholars have verified that clock modelling improves the accuracy of height direction [8,15,16]. As demonstrated by the abovementioned work, showing two state clock models involving WFM and RWFM noise that are frequently employed in Kalman filter covariance. The flicker noise is considered to be between random walk noise and white noise, and plays an important role at low offset frequencies, but it has been generally approximated in the existing model. It is impossible to model flicker noise reliably and exactly in a finite order state model [17]. The three-state clock model, in addition to estimate the clock bias and frequency bias, the frequency drift has been estimated in the model, some valuable literatures has been published about the three-state clock model [18,19].

In this contribution, the stochastic model with three kinds of noise has been exploited in the PPP solution to achieve better timing accuracy. Considering the diversification of settings, the timing demand is not only in the static field but also in the kinematic field. We first provide some descriptions of the concept of the clock model and then present experimental data and processing strategies. In addition, we validate the feasibility of the clock model in satellite timing with a static scheme and kinematic scheme. In the subsequent part, the results obtained with multiple schemes are demonstrated. The conclusions are drawn in the final section.

## 2. The Feasibility of a Two-State Clock Model with Three Kinds of Noise for Use in a PPP Kalman Filter Estimation

Initially, clock offset is considered in the Kalman filter to be the simplest model and allows for arbitrary increments by adding large amounts of noise during the covariance update [20]. This scheme is reasonable because extensive process noise can account for all variations in the clock offset. In a short time, a two-state clock is put into practice, the expression of which is physically meaningful. On the basis of the two-state clock model, frequency drift is considered and results in the generation of a three-state clock model, which is not practical because the filter process is complicated and time-consuming [21]. Therefore, we focused our attention on the two-state clock model.

As a rule, a clock signal is mainly affected by white frequency noise, flicker frequency noise, and random walk frequency noise. The different noise variances can be obtained according to the integration of the noise coefficients:

(1) $$\begin{array}{c} h_{y_{0}}^{}(t)=\sqrt{h_{0}/2}\delta (t);h_{y_{-1}}^{}(t)=\sqrt{h_{-1}/t};h_{y_{-2}}^{}(t)=\pi \sqrt{2h_{-2}}l(t);\\ h_{x_{0}}^{}(t)=\sqrt{h_{0}/2}l(t);h_{x_{-1}}^{}(t)=2\sqrt{h_{-1}t};h_{x_{-2}}^{}(t)=\pi \sqrt{2h_{-2}}t;\\ h_{y_{0}}^{}(t),h_{y_{-1}}^{}(t),h_{y_{-2}}^{}(t),h_{x_{0}}^{}(t);h_{x_{-1}}^{}(t);h_{x_{-2}}^{}(t) \end{array}$$

represents the respective impulse responses. Where *h*_0_, *h*_−1_ and *h*_−2_ denote the white frequency noise, flicker frequency noise, and random walk frequency noise, respectively, and *δ*(*t*) is the Dirac delta function, and *l*(*t*) is the unit response function; that is:

(2) $$\delta (t)=\{\begin{array}{cc} 1 & \tau =0\\ 0 & \tau >0 \end{array},\;l(t)=1$$

The cross-correlation function between the two processes is:

(3) $$\sigma_{}^{2}(t)=\int_{0}^{t}h_{x}(u)h_{y}(u+\tau )du;\tau \geq 0$$

With Equation (3), three kinds of variance employed in the two-state filter are obtained as follows:

(4) $$\begin{array}{l} \sigma_{x_{-1}}^{2}(t,\tau )=\int_{0}^{t}h_{x_{-1}}(t)h_{x_{-1}}(t+\tau )dt=\int_{0}^{t}2\sqrt{h_{-1}t}2\sqrt{h_{-1}(t+\tau )}dt\approx (2t+\tau )h_{-1}\sqrt{t(t+\tau )}+\frac{\tau^{2}}{2}\ln\tau h_{-1}\\ \sigma_{x_{-2}}^{2}(t,\tau )=\int_{0}^{t}h_{x_{-2}}(t)h_{x_{-2}}(t+\tau )dt=\int_{0}^{t}\pi \sqrt{2h_{-2}}t\pi \sqrt{2h_{-2}}(t+\tau )dt=2\pi^{2}h_{-2}(\frac{t^{3}}{3}+\frac{\tau t^{2}}{2})\\ \sigma_{y_{0}}^{2}(t,\tau )=\int_{0}^{t}h_{y_{0}}(t)h_{y_{0}}(t+\tau )dt=0\\ \sigma_{y_{-1}}^{2}(t,\tau )=\int_{0}^{t}h_{y_{-1}}(t)h_{y_{-1}}(t+\tau )dt=\int_{0}^{t}\left(\sqrt{\frac{h_{-1}}{t}}\sqrt{\frac{h_{-1}}{t+\tau }}\right)dt\approx -\ln\tau h_{-1}\\ \sigma_{y_{-2}}^{2}(t,\tau )=\int_{0}^{t}h_{y_{-2}}(t)h_{y_{-2}}(t+\tau )dt=\int_{0}^{t}{\left(\pi \sqrt{2h_{-2}}\right)}^{2}dt=2h_{-2}\pi^{2}t\\ \sigma_{x_{0}y_{0}}^{2}(t,\tau )=\int_{0}^{t}h_{x_{0}}(t)h_{y_{0}}(t+\tau )dt=\int_{0}^{t}{\left(\pi \sqrt{2h_{-2}}\right)}^{2}dt=0\\ \sigma_{x_{-1}y_{-1}}^{2}(t,\tau )=\int_{0}^{t}h_{x_{-1}}(t)h_{y_{-1}}(t+\tau )dt=\int_{0}^{t}2\sqrt{h_{-1}}t\sqrt{\frac{h_{-1}}{t+\tau }}dt\approx 2h_{-1}(t+\tau )\sqrt{\frac{t}{t+\tau }}+2h_{-1}\tau \ln\sqrt{\tau }\\ \sigma_{x_{-2}y_{-2}}^{2}(t,\tau )=\int_{0}^{t}h_{x_{-2}}(t)h_{y_{-2}}(t+\tau )dt=\int_{0}^{t}{\left(\pi \sqrt{2h_{-2}}\right)}^{2}dt=\pi^{2}h_{-2}t^{2} \end{array}$$

Here, the variance and covariance are obtained by integration. According to the error propagation law, the process noise cofactor matrix is:

(5) $$\begin{array}{l} \mathrm{cov}(x(t),y(t))=\\ \left[\begin{array}{cc} \frac{h_{0}}{2}t(2t+\tau )+h_{-1}\sqrt{t(t+\tau )}+\frac{\tau^{2}}{2}\ln\tau h_{-1}+2\pi^{2}h_{-2}(\frac{t^{3}}{3}+\frac{\tau t^{2}}{2}) & 2h_{-1}\sqrt{t(t+\tau )}+2h_{-1}\tau \ln\sqrt{\tau }+\pi^{2}h_{-2}t^{2}\\ 2h_{-1}\sqrt{t(t+\tau )}+2h_{-1}\tau \ln\sqrt{\tau }+\pi^{2}h_{-2}t^{2} & -\ln\tau h_{-1}+2h_{-2}\pi^{2}t \end{array}\right] \end{array}$$

By setting t=τ, we obtain:

(6) $$\left[\begin{array}{cc} \frac{3h_{0}t^{2}}{2}+\sqrt{2}h_{-1}t+\frac{t^{2}\cdot \ln t\cdot h_{-1}}{2}+\frac{5\pi^{2}h_{-2}t^{3}}{3} & 2\sqrt{2}h_{-1}t+2h_{-1}t\ln\sqrt{t}+\pi^{2}h_{-2}t^{2}\\ 2\sqrt{2}h_{-1}t+2h_{-1}t\ln\sqrt{t}+\pi^{2}h_{-2}t^{2} & -\ln t\cdot h_{-1}+2h_{-2}\pi^{2}t \end{array}\right]$$

The modified variance is employed for the estimation of cofactors *h*_0_, *h*_−1_ and *h*_−2_. The noise coefficients can be obtained with least squares via different averaging times with the following equation:

(7) $$\sigma_{y}^{2}(\tau )=\;\frac{2\pi^{2}h_{-2}}{3}\tau +2\ln2\cdot h_{-1}+\frac{h_{0}}{2}\cdot \frac{1}{\tau }+\frac{h_{1}[6+3\ln(2\pi f_{h}\tau )-\ln2]}{4\pi^{2}}\cdot \frac{1}{\tau^{2}}+\frac{3f_{h}h_{{}^{2}}^{}}{4\pi^{2}}\cdot \frac{1}{\tau^{2}}$$

White noise is the main function in the effective time, which is decided by the physical characteristic of the atomic clock. The phase noise spectral density is transformed into a time domain covariance model that can be used to derive the Kalman filter model parameters. Hence, three kinds of noise have been adopted in this work. The modelling employed for the Kalman filter is based on adding the relative constraints on adjacent receiver clock offsets and degrading the correlation between the receiver clock and other parameters. Of course, the noise coefficients are not unique and can be obtained by different averaging times. The influence induced by the coefficient discrepancy is negligible and can be ignored.

## 3. Analysis of Position and Troposphere with Clock Modelling

### 3.1. Data Description

To validate the proposed stochastic clock model employed in the PPP dual-frequency ionosphere-free approach, both static and kinematic experiments were conducted. The observation data of IGS stations for 18 days (days of the year (DOY) 91 to 109 in 2020) were selected. The data of 18-days is much enough to be certified the superiority of modelling. The precise clock and orbit products are provided by IGS. The file igs14.atx was used to correct GPS phase centre offsets (PCOs) and phase centre variations (PCVs). The relative information is listed in Table 1.

The PPP processing strategies are listed in Table 2. All kinds of influences on the signal must be corrected or modelled with the required precision. Elevation-dependent weighting is applicable in PPP applications and can compensate for the deficiencies of troposphere modelling at low elevations.

### 3.2. The Feasibility of Clock Modelling

**Figure 1:**
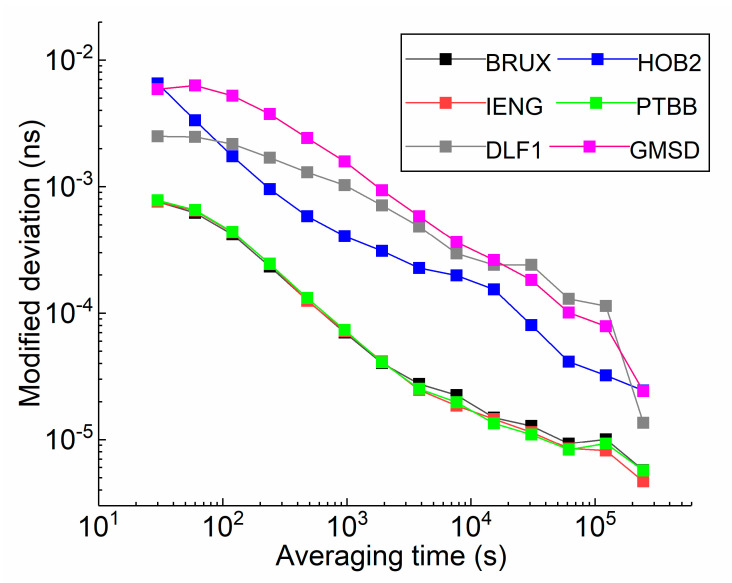
The modified deviation of a receiver clock offset estimated by GPS PPP estimation.

The modified deviation of the receiver clock time series is shown in Figure 1 and is different for each station. It is regarded as a reliable indicator for evaluating clock quality. The receiver clock offset is the difference between the satellite navigation system time and the local clock time. The actual clock behaviour is not affected by the system time because of the sufficiently stable IGS time scale. As shown in Figure 1, in the hydrogen maser, the modified deviations for the BRUX, IENG, and PTBB clock offsets are similar and can reach a frequency stability of 7 E-13 at an averaging time of 30 s or better, while the stability of the HOB2 clock offset is almost one order of magnitude higher and merely reaches a frequency stability of 6 E-12 at the same averaging time. For the caesium clock, the modified deviations for DLF1 and GMSD are similar and lower than 1 E-12 when the averaging time is fixed at 30 s. Next, the clock is divided into two groups for future analysis: the clocks of BRUX, IENG, and PTBB belong to the ‘good’ clock group, and the others can be classified in the ‘bad’ clock group.

Notably, the noise coefficients have been provided in previous studies; in fact, it has been verified that the empirical values are not the optimal choice for PPP position accuracy [26].

**Figure 2:**
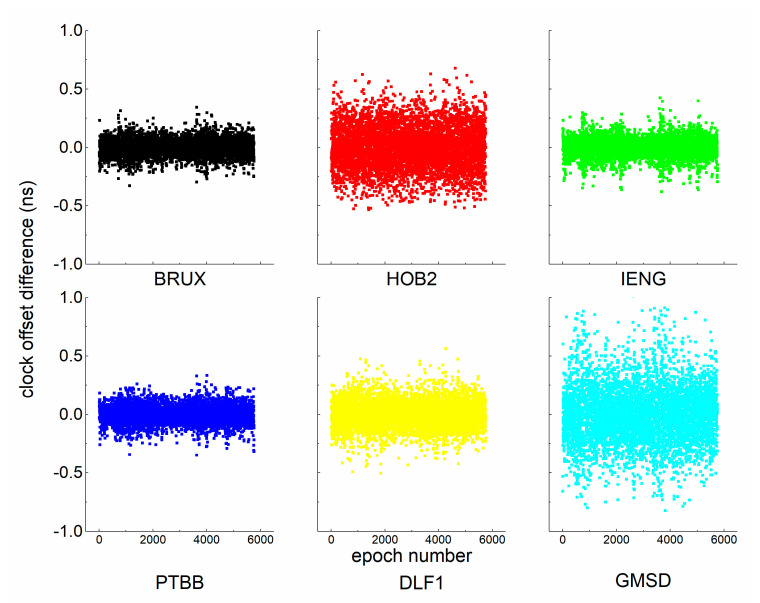
Between-epoch clock offset differences at four stations compared to the IGS final products.

Unfortunately, more useful information concerning stable clocks has been wasted, as shown in Figure 2. We see that the between-epoch clock offset differences are small and stable. Based on Equation (7), a suite of coefficients was obtained with one day of observation. The noise coefficients involved in this study are listed in Table 3.

### 3.3. The Analysis of Correlation Coefficient

All the satellites were observed above the horizon, resulting in a strong correlation between the receiver clock estimates and the height coordinate estimates, which leads to degraded position accuracy. With modelling, the correlation is varied in each pair of estimates in the PPP solution. To qualify the extent of the decorrelation, the correlation coefficients are obtained according to Equation (8):

(8) $$\rho =\frac{\mathrm{cov}(\delta a,\delta b)}{\sqrt{\sigma_{\delta a}^{2}\cdot \sigma_{\delta b}^{2}}}$$

where ρ represents the correlation coefficient, cov(δa,δb) are the covariances, and $\sigma_{\delta a}^{2}$ and $\sigma_{\delta b}^{2}$ are the variances. cov(δa,δb), $\sigma_{\delta a}^{2}$ and $\sigma_{\delta b}^{2}$ are obtained from the updated estimate covariance matrix of the Kalman filter.

**Figure 3:**
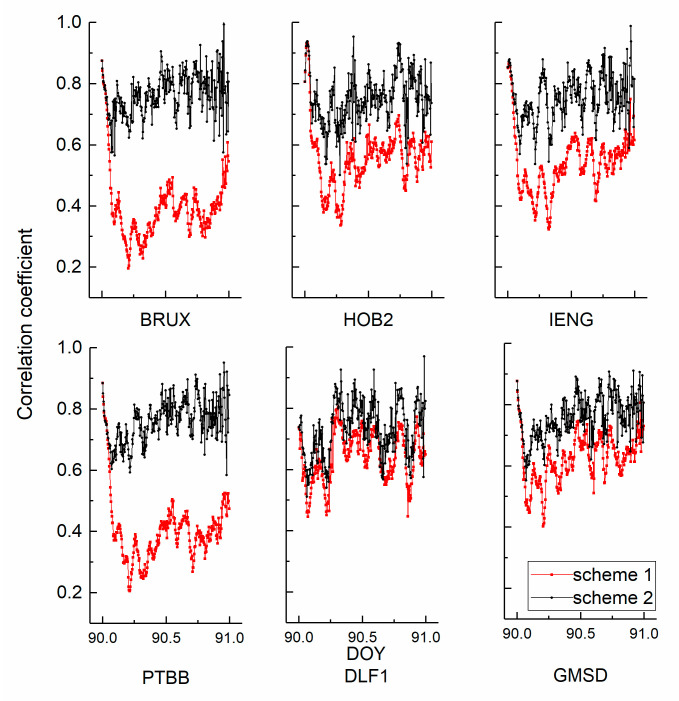
The correlation coefficients between the kinematic receiver clock offsets and height position estimates.

**Figure 4:**
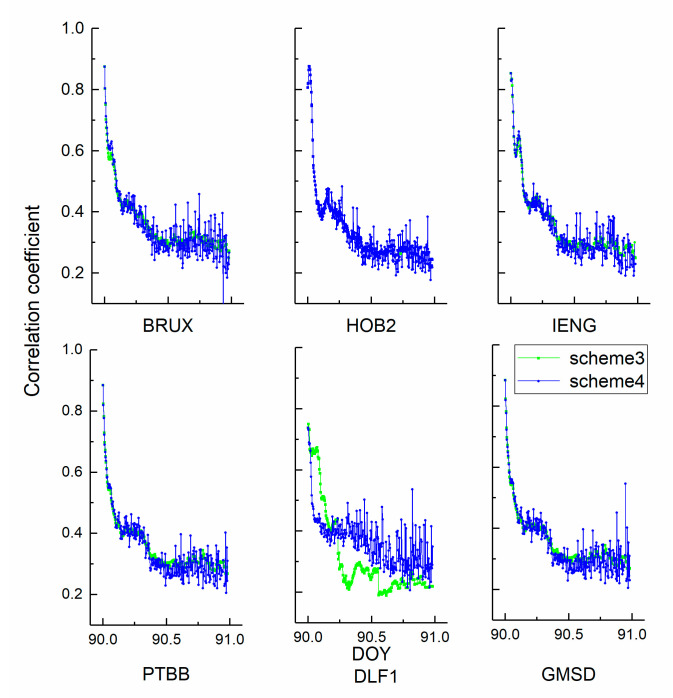
The correlation coefficients between the static receiver clock offsets and height position estimates.

Figure 3 and Figure 4 show the correlation coefficients between the height position and receiver clock estimates in kinematic and static PPP modes. Red, black, green, and blue represent schemes 1, 2, 3, and 4, respectively. Three general conclusions can be made. First, the coefficient variations of the kinematic scheme are more evident than those of the static scheme. The average coefficients of the kinematic solution decrease from 0.8 to 0.3. Second, the curves show a visible hierarchy between scheme 1 and scheme 2, while scheme 3 and scheme 4 do not. In addition, the decorrelation degree induced by the hydrogen maser is more obvious than that of the caesium clock. Returning to Figure 1, the stability of the hydrogen maser is better than that of the caesium clock. Third, the values of the static coefficients are smaller than those of the kinematic scheme, which keeps the number at a level of 0.3. The position of the kinematic scheme is unknown in each epoch.

### 3.4. Analysis of Position Performance

**Figure 5:**
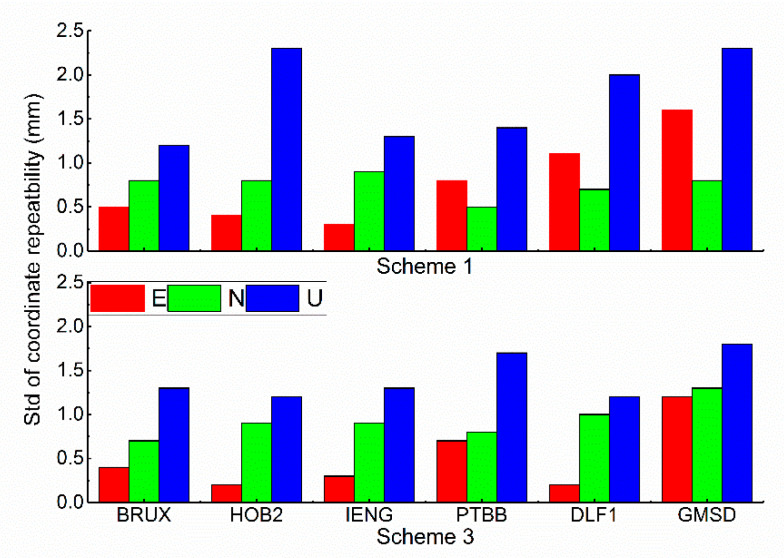
The standard deviation of coordinate repeatability for the six stations.

**Figure 6:**
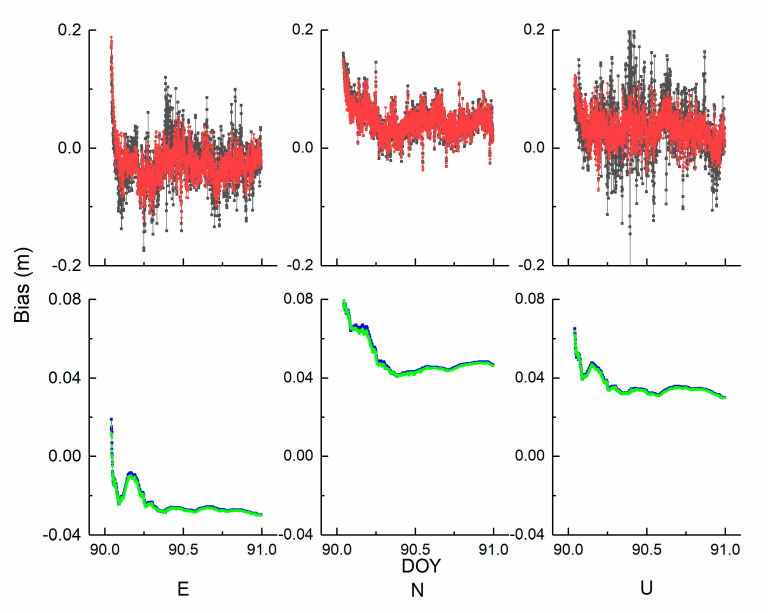
The time series of BRUX East (E), North (N), and Up (U) positioning accuracy.

The standard deviations of coordinate repeatability for the six stations are demonstrated in Figure 5. The analysis of position performance and the external reference station coordinates are missing. If station coordinates with higher accuracy are derived, then the repeatability data may be more suitable than the reference coordinates. The standard deviations of the kinematic scheme is larger than that of the static scheme. The standard deviations in the U direction are larger than those in the E and N directions. The whole standard deviation is less than 2.5 mm. The biases of the coordinates in the north, east, and up directions between the estimated values and true values are demonstrated in Figure 6. The colour code is the same meaning as it is in Figure 4. The station BRUX was randomly selected to validate whether the position accuracy is improved with the auxiliary clock modelling. At first glance, in the kinematic mode, the results with clock modelling demonstrate a more concentrated tendency than those without clock modelling. In contrast, there is no obvious visual difference in the static scheme. To inspect the underlying benefits of clock modelling, we quantify the results in terms of the root mean square (RMS). The E, N, and U RMS values of schemes 1 and 2 are 0.040 m and 0.045 m, 0.041 m and 0.054 m, and 0.041 m and 0.055 m, respectively. The improvements in kinematic position are 11.11, 24.07, and 25.45%. In addition, the E, N, and U RMS values of schemes 3 and 4 are 0.024 m and 0.025 m, 0.035 m and 0.037 m, and 0.048 m and 0.052 m, respectively, and the improvements in the static positions are 4.17, 5.41, and 7.69%, respectively. Notably, the vertical direction is evidently enhanced relative to the horizontal direction because of the clock information. The improvement magnitude of schemes 1 and 2 is remarkably better than that of schemes 3 and 4. Two kinds of position schemes can both reach the cm level. Incorporating the clock information, as the kinematic mode is concerned, the relationship is no longer loose in the neighbouring epoch. In other words, there is no relationship between adjacent epochs. In contrast, the position is invariable in the static solution. The data show that a proper clock modelling strategy is preferred for the kinematic solution.

### 3.5. The Analysis of Troposphere Performance

**Figure 7:**
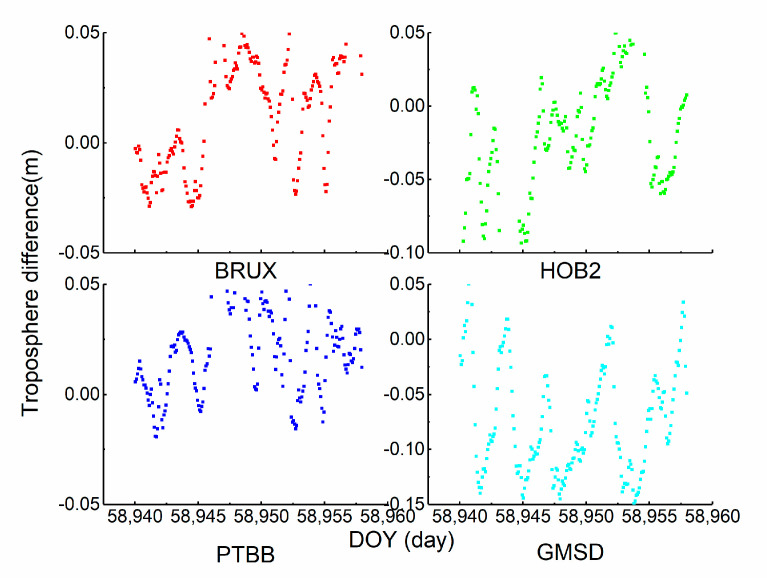
The time series of zenith path delay parameters (ZPD).

It is shown that the receiver clock offsets, vertical component of coordinate, and ZPD are strongly correlated; therefore, in Section 3.3, the analysis of the correlation between the receiver clock offset and vertical component is displayed. To verify the accuracy of the ZPD value estimated by the proposed method, the difference between the IGS ZPD product and the estimated value of scheme 3 is exhibited in Figure 7. The four stations were selected for the comparison. The sampling of the IGS ZPD product is 7200 s. The results show that the RMS values of the four stations are 0.03, 0.05, 0.03, and 0.08 m. The negligible discrepancy between these values indicates that the troposphere accuracy cannot be influenced by clock modelling.

## 4. The Evaluation of Timing Performance

**Figure 8:**
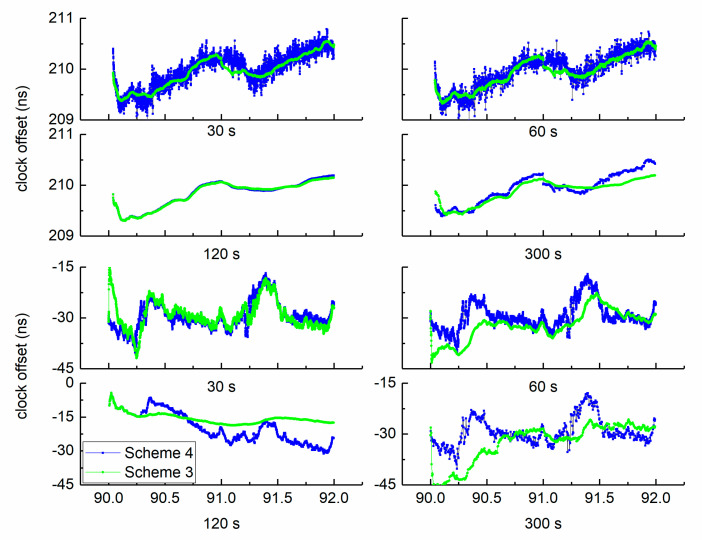
The clock offset of stations BRUX and GMSD on DOY 90–91.

To investigate the influence of sampling time on the model, the results estimated by different sampling times were obtained and compared. In Figure 8, taking the static scheme as an example, the upper two rows are from station BRUX, and the latter two rows are from station GMSD. In terms of the ‘good’ clock, when the sampling is 30 s or 60 s, the clock offset generated with the clock modelling seems less noisy and smoother than the result obtained with the white noise. When the sampling is 120 s or even longer, clock modelling is disabled. Considering station GMSD, the model works only in the sampling of 30 s. Improper results are obtained when large sampling is employed. We can explain that the variation of the ‘good’ clock is steady over a relatively large interval; therefore, the behavior of atomic clocks can be accurately described by the model.

**Figure 9:**
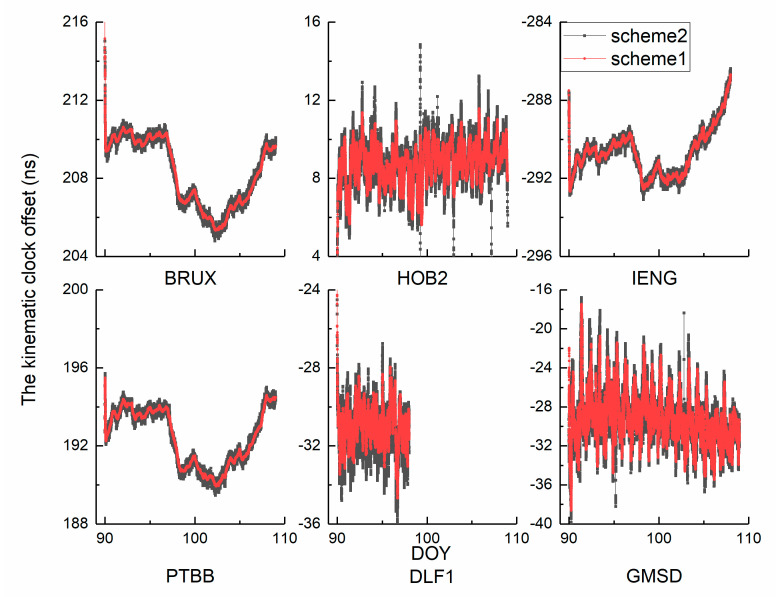
The kinematic clock offset time series of GPS PPP.

**Figure 10:**
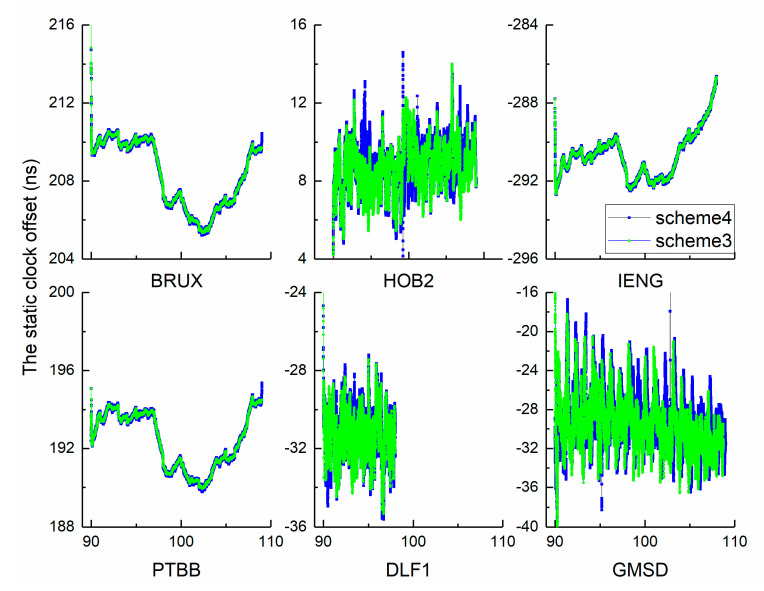
The static clock offset time series of GPS PPP.

As the sampling time increases to 30 s, 60 s, 120 s, and 300 s, the number of parameters decreases. Therefore, inaccurate results are not related to overparameterization. Figure 9 and Figure 10 show two time series. Theoretically, the clock offset is dominated by white noise, which disperses near zero In fact, the results include the receiver hardware delay. The calibration issues can be neglected in the following analysis because of the stability of the receiver hardware delay. In the investigation period, complete observations of station DLF1 were selected from DOY 90 to 98. Overall, the two curves of each independent panel show strong similarities, which indicates the reasonability of our approach. Simultaneously, an important hint has been identified from the full views. We observed that the results of schemes 1 and 3 are more dispersed than those of schemes 2 and 4, which indicates that the noise of the clock offset time series has been weakened by clock modelling in the kinematic mode as well as in the static mode. To further quantify the advantage of clock modelling, the modified deviation was calculated for statistical analysis.

**Figure 11:**
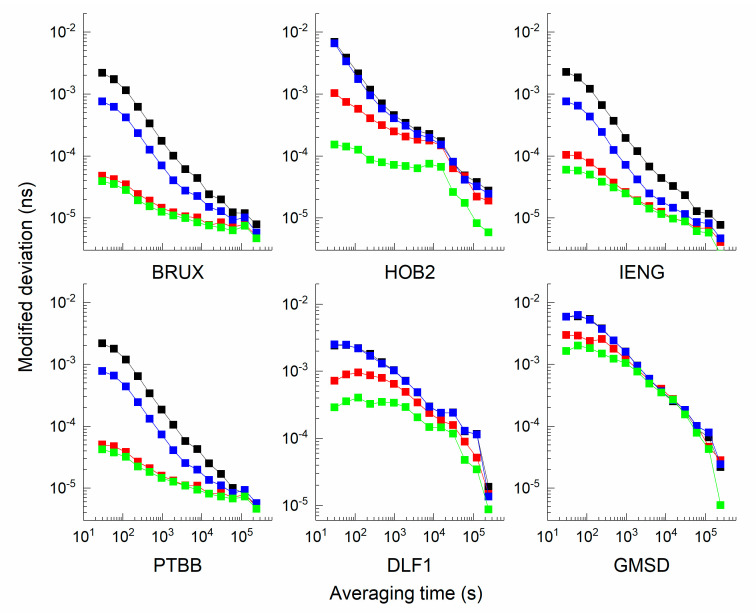
Comparison on modified deviation of six stations.

**Figure 12:**
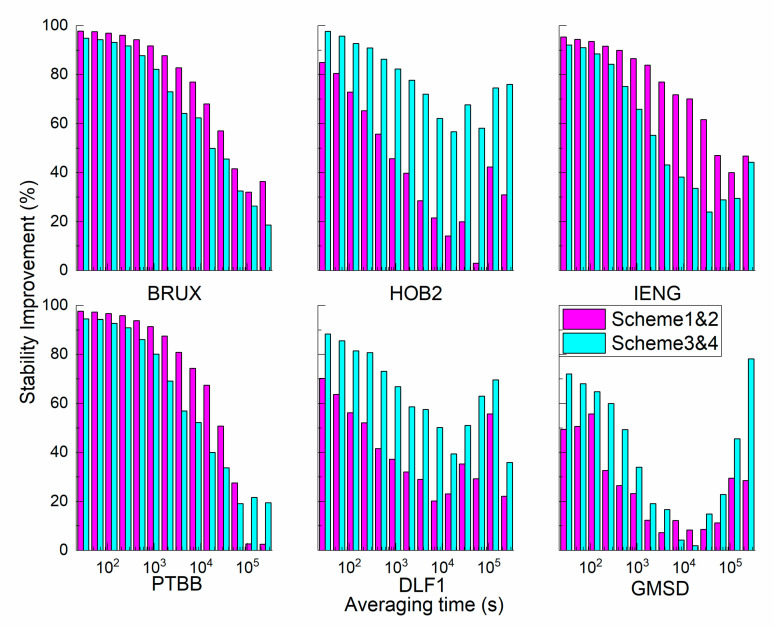
The improvement percentage of the stability of the GPS receiver clock offset with and without the clock model.

To determine the potential of clock modelling in timing, the modified deviation is presented in Figure 11, and the improvement percentage of stability is compared in Figure 12. It can be observed that the stability of the static scheme is more stable than that of the kinematic scheme; specifically, the stability of the scheme incorporating modelling is more stable than that without clock modelling. To determine whether the improvement depends on the clock type and averaging time, the analysis was conducted in two ways. For stations BRUX, IENG, and PTBB, the improvement of the kinematic scheme is larger than that of the static scheme; however, for stations HOB2, DLF1, and GMSD, the percentage value of the static scheme is larger than that of the kinematic scheme. The discrepancy is related to the clock type: the model works well for the ‘good’ clock in the kinematic mode, while the model is preferable for the ‘bad’ clock in the static mode.

When the receiver moves, the position variance has been initialized at each epoch, and no prior coordinate information can be propagated into the current calculation. Under free conditions, the induction of clock modelling is equivalent to a new constraint for the filter equation to shorten convergence time and estimate more accurate parameters. However, in static mode, all prior information is available for parameter estimation. Now, regarding the clock type, the behaviour of the ‘good’ clock can be accurately described by the noise composition, while the ‘bad’ clock is not. That is, the additional information of the ‘bad’ clock imposes no effect on the final results. Another point of interest is the averaging time. Compared with long-term stability, the model is more accurate for short-term stability.

As shown in Table 4, the following findings are evident: (1) for all stations, the maximal value is 96.98%, and the minimal value is 2.97%. The average values of the static scheme and kinematic scheme are 53.53% and 59.11%, respectively. (2) For all averaging times, the improvement to short-term stability is more obvious than the improvement to the long-term stability. When the averaging time ranges from 100 s, 1000 s, and 10000 s to 86400 s, the average values of the static scheme are 78.72, 62.64, 46.17, and 26.62%, and the average values of the kinematic scheme are 85.57, 68.55, 44.89, and 37.42%. With increased sampling time, the extent of the improvement weakens. For example, at the BRUX station, the average percentages of the static scheme are 96.98, 91.77, 76.96, and 41.58%, and the average percentages of the kinematic scheme are 93.27, 82.17, 62.39, 32.50, and 64.41%, respectively. (3) For the ‘good’ clock, the average percentages of the static scheme are 95.77, 89.91, 74.36, and 38.74%, and the average percentages of the kinematic scheme are 91.47, 76.06, 50.94, and 26.84%. For the ‘bad’ clock, the average percentages of the static scheme are 61.66, 35.36, 17.98, and 14.50%, and the average percentages of the kinematic scheme are 79.66, 61.04, 38.84, and 48.00%, respectively.

**Figure 13:**
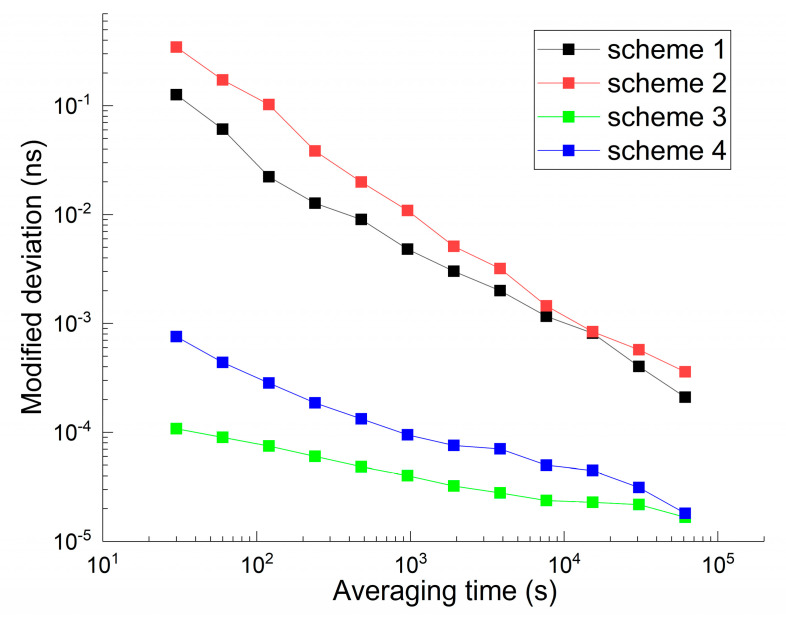
The stability improvement of BDS-3 time transfer with four schemes.

To verify the universality of clock modelling with other GNSSs, BDS was employed in this experiment. Of all the stations, BRUX and PTBB stations can receive BDS-3 signals. These two stations are timing laboratories located in Europe. B1I and B3I signals contribute to the calculation. Hence, the link BRUX-PTBB BDS-3 time transfer is displayed in Figure 13. As expected, clock modelling is also suitable for BDS-3 time transfer, which leads to the same conclusions as those obtained with GPS: the stability of the time series considering clock modelling is better than that of the common solution, and the stability of the static scheme is superior to that of the kinematic scheme.

## 5. Conclusions

Accompanied by the enhanced performance of atomic clocks, the application of an appropriate clock model becomes possible. In this study, the relation between the clock type, sampling time and the clock modeling is investigated by virtue of the static scheme and kinematic scheme. The results of the four kinds of strategies were compared.

Several findings are summarized in the following:

(1) Whatever the kinematic or static scheme used, the timing stability has been remarkably enhanced at all the averaging time, especially, it is effective for the short term stability. Furtherly, the stability improvement has some relation with the clock type and the processing strategy. As ‘good’ clock as concerned, the frequency stability improvement of kinematic scheme is bigger than that of the static scheme. For the ‘bad’ clock, the stability improvement of kinematic scheme is smaller than that of the static scheme.
(2) When the clock modeling is applied, the correlation between the height component and the receiver clock offset has been degraded. The decorrelation extent of kinematic solution is more obvious than that of static scheme.
(3) The application of clock modeling is related to the sampling and the clock type. The good clock is preferred for clock modeling. When the sampling goes longer, the model is possible to useless.

## Figures and Tables

**Table 1:** Station information involved in the experiment.

Station Name	Location	Receiver Type	Antenna Type	External Clock
BRUX	Belgium	SEPT POLARX5TR	JAVRINGANT_DM	Active H-MASER
HOB2	Austria	SEPT POLARX5	AOAD/M_T	H-MASER
IENG	Italy	SEPT POLARX4TR	SEPCHOKE_C	H-MASER
PTBB	Germany	CETC-54-GMR-4016	LEIAR25.R4	H-MASER
DLF1	The Netherlands	TRIMBLE NETR9	LEIAR25.R4	CESIUM
GMSD	Japan	TRIMBLE NETR9	TRM59800	CESIUM

**Table 2:** PPP processing strategies.

Item	Strategies
Estimator	Kalman filter
Relativistic effect	IERS conventions 2010 [22]
Sagnac effect	IERS conventions 2010
Phase wind-up	Model corrected to wu [23]
Ionospheric delay	IF linear combination
Tide displacements	IERS conventions 2010
Tropospheric delay	estimated as a continuous piecewise linear function (2 h parameter spacing) [24]
Receiver clock offset	Estimated via the white noise process [25]
Ambiguity	Estimated as a constant
Station coordinate	Static solution: estimated as a constant
	Simulated kinematic solution: estimated as white noise
Solution	Scheme 1 Kinematic mode with clock model
	Scheme 2 Kinematic mode with white noise
	Scheme 3 Static mode with clock model
	Scheme 4 Static mode with white noise

**Table 3:** The noise coefficients of the external clock connected with IGS stations.

IGS Station	h-2	h-1	h-0
BRUX	2.52 e-29	−1.05 e-25	8.22 e-23
HOB2	−2.13 e-29	1.52 e-25	1.05 e-23
IENG	1.76 e-29	−7.89e-26	6.21 e-23
PTBB	2.40 e-29	−1.00 e-25	7.63 e-23
DLF1	−1.58 e-27	3.33 e-24	3.83 e-22
GMSD	1.13 e-27	−7.37 e-24	1.38 e-20

**Table 4:** Improvement percentage of stability of the GPS receiver clock offset with and without the clock modelling (static: sta; kinematic: kin).

	Station	BRUX		HOB2		IENG		PTBB		DLF1		GMSD	
Averaging Time (s)		sta	kin	sta	kin	sta	kin	sta	kin	sta	kin	sta	kin
100		96.98	93.27	72.96	92.74	93.58	88.47	96.76	92.68	56.26	81.46	55.76	64.80
1000		91.77	82.17	45.65	82.33	86.58	65.86	91.40	80.16	37.23	66.89	23.22	33.92
10,000		76.96	62.39	21.46	62.14	71.80	38.19	74.32	52.26	20.24	50.16	12.24	4.22
86,400		41.58	32.50	2.97	58.14	47.02	28.94	27.62	19.09	29.27	63.05	11.27	22.82

## Data Availability

All data generated or appeared in this study are available upon re-quest by contact with the corresponding author.
